# Preparation, Emulsion Stability and Preliminary Demoulding Evaluation of a Wax-Free Water-Based Soybean Oil-Mineral Oil Concrete Release Agent

**DOI:** 10.3390/nano16211429

**Published:** 2026-11-05

**Authors:** Biliang Zhang, Jiejun Yuan, Haidong Zhang, Wei Xu, Yongmei Wang, Leiying Han, Xiaoyan Liu

**Affiliations:** 1 Jiangsu Power Transmission and Transformation Co., Ltd., Nanjing 210000, China; 2 College of Civil Engineering and Transportation, Hohai University, Nanjing 210098, China

**Keywords:** concrete release agent, emulsion, soybean oil, sodium benzoate, demoulding force

## Abstract

Water-based, wax-free release agents are attractive for concrete formwork, yet their emulsification window and demoulding behaviour remain poorly defined. This work prepared a soybean-oil/naphthenic-oil emulsion and evaluated its stability and pull-off performance. OP-10/Span-80 blends produced a visually stable soybean-oil emulsion in a narrow hydrophilic–lipophilic balance (HLB) window, with only trace or small separation within 1 h across a calculated HLB interval of 12.15–12.31 at an assumed OP-10 HLB of 14.5 (11.3–12.3 across the reported range). The high-non-volatile-content naphthenic-oil emulsion containing 1.00 wt% sodium benzoate (MH2+) showed no visible phase boundary after centrifugation at 7000 rpm for 6 min and only a thin re-emulsifiable surface layer after 20 d, whereas the sodium-benzoate-free emulsion separated into three phases. At 1.57–1.59 micrometres, the 1:3 blend of the soybean-oil emulsion and MH2+ gave a pull-off force of 7.92 N, comparable to EP6 (7.963 N at 1.50 micrometres) and lower than MH2+ (11.63 N) and the soybean-oil emulsion (55.26 N), and produced visually more uniform demoulded surfaces. The blend force increased to 73.68 N at 1.05 micrometres, indicating that sufficient coating is essential. The 1:3 blend is therefore a promising low-force, wax-free water-based release agent at sufficient coating, providing a basis for coating optimization and field validation.

## 1. Introduction

Formwork is a central component of concrete construction [1], and as architectural design and concrete technology advance, the construction industry has set increasingly high standards for decorative concrete surfaces, making release-agent development an important topic in civil and hydraulic engineering [2]. Release agents reduce adhesion and friction between formwork and concrete, helping prevent surface damage during demoulding [3,4]. In hydraulic structures, erosion-resistant concrete is used on dam faces; pitting and blowholes can intensify long-term flow erosion and allow harmful substances to enter concrete. Appropriate release agents can improve surface appearance and may support durability [5,6]. Paraffin and cycloalkane derivatives, as well as mineral, lubricating, vegetable and reclaimed oils, are used in commercial formulations, and formwork type together with release-agent selection affects near-surface density, durability-related indicators and surface appearance [7]. Storage instability and sedimentation can nevertheless create surface defects. Solvent-based agents are flammable and create health and environmental risks; water-based alternatives reduce solvent use but frequently contain wax, which may affect formwork service life [8]. Film formation from a waterborne coating depends on droplet concentration, coalescence and spreading [9], and drying studies of oil-in-water emulsions on substrates show that substrate wettability influences wetting and the distribution of the residual film [10]. The type of release oil can also influence steel-formwork corrosion and facing esthetics [11]. Emulsifier selection also controls emulsion stability [12]. Rain exposure adds a separate washout and retention problem that standard release tests do not establish. Formwork surface energy, texture and friction against fresh concrete further influence demoulding [13,14,15]. A wax-free water-based formulation therefore merits evaluation under controlled laboratory conditions, with storage stability, toxicity and rain resistance treated as separate performance dimensions. Libessart et al. [16] developed a release agent from used cooking oil; Lu et al. [17] evaluated the release performance of a concrete release agent; de Brito et al. [18] evaluated the technical performance of vegetable-oil-based release agents; and Libessart et al. [19] compared the tribological behaviour of emulsions and demoulding oils at the concrete/formwork interface.

To examine technical feasibility, this study investigated soybean-oil emulsification, the stability of the MH2 mineral-oil emulsion, and the preliminary release behaviour of a 1:3 soybean-oil emulsion/MH2+ blend. The objective was to test the formulation and workflow, not to demonstrate field durability or environmental superiority. The formulation retains the practical characteristics of a water-based release agent, but its engineering applicability still requires field validation.

Research gap and contribution: The principal gap addressed here is not the isolated use of vegetable oil, mineral oil or sodium benzoate, because these components have been examined separately [16,17,18,20]. Abbas et al. reported an ultrastable oil-in-water concrete release agent [3]. The present study asks whether a high-non-volatile (about 56 wt%) soybean-oil/naphthenic-oil formulation can be prepared in a low-speed laboratory mixer with 1 wt% sodium benzoate and oleic acid, and whether its wet-state behaviour can be connected to film distribution and pull-off force. The contributions are: (i) an experimentally constrained, emulsifier-specific HLB window; (ii) a feasible low-speed laboratory preparation route, with energy consumption treated as a separate scale-up question; (iii) a workflow linking formulation screening and visual/centrifugal stability to pull-off force; and (iv) an explicit boundary between observations, plausible mechanisms and claims requiring field testing.

The claim is therefore not that any individual component is used for the first time. The most defensible contribution is the combination of a specific high-non-volatile mixed-oil formulation, a low-speed laboratory preparation condition, and a quantitative workflow linking emulsion state to film behaviour and pull-off force. Colour uniformity is not presented as a demonstrated innovation. The comparison with Abbas et al. defines the scope rather than claiming superior performance. The lower stirring speed is reported only as a feasible processing condition because the available trials also changed composition, oil fraction, emulsifier loading and additives.

Study organization: The work comprised four parts: (1) HLB and emulsifier screening for soybean oil; (2) emulsification equipment and process trials; (3) qualitative retention tests of base oils on formwork materials; and (4) stability and preliminary pull-off evaluation of MH2, MH2+, and their 1:3 blend. The study is positioned as an exploratory formulation and measurement-workflow study; no inferential statistics are drawn from a single storage batch or from two force records per condition.

## 2. Materials

Cycloalkane Oil 4010, Jinlongyu Grade 1 Soybean Oil, Oleic Acid, Span-80, OP-10, Tween-80, CO-40, EL-40, SDS, Tripolyglycerol Monostearates, Tripolyglycerol Monooleate, Decapolyglycerol Monooleate, Soybean Lecithin (60%), Wetting Agent Dow CF-10, Sodium Benzoate. The experimental emulsifiers and their properties are summarized in Table 1.

## 3. Methods

Step 1: Using the hydrophilic–lipophilic balance (HLB) method [21], we emulsified soybean oil using Span-80 (HLB = 4.3) and OP-10 (HLB = 13.3~14.5) in combination with other emulsifiers of varying HLB values, and the HLB value yielding the best stability was determined through observation.

Formulations and nomenclature: Unless stated otherwise, all formula percentages are mass fractions of the prepared emulsion. MH2 contained 48.36 wt% 4010 naphthenic oil, 2.96 wt% oleic acid, 2.14 wt% OP-10, 2.14 wt% Span-80 and 44.40 wt% deionized water. MH2+ contained the same oil and emulsifier amounts plus 1.00 wt% sodium benzoate; water was reduced to 43.40 wt%, comprising 41.11 wt% added directly and 2.29 wt% used to dissolve the sodium benzoate. The soybean-oil emulsion replaced the naphthenic oil with 48.36 wt% soybean oil and otherwise used the corrected composition above. All corrected formulations sum to 100.00 wt%. The 1:3 sample was prepared by mixing the two finished emulsions at 1:3 by mass; it is not a ratio of neat oil components.

Preparation and process controls: Four process variants were used for different experiments and are not pooled as direct replicates: (1) the initial HLB screen used 20 g soybean oil, 5 g blended emulsifier and 80 g water at 4000 rpm, with water added over 20 min and another 20 min of dispersion; (2) the homogenizer/disperser comparison used 19% soybean oil, 4.8% blended emulsifier and 76% water at 4000 rpm for 5 min, followed by 20 min of water addition and 20 min of dispersion; (3) the high-oil-phase batch used 48.36/4.28/2.96/1.00/43.40 wt% oil/emulsifier/oleic acid/sodium benzoate/water at 600 rpm for 10 min, with water and sodium-benzoate solution added over a combined 45 min and dispersion at 800 rpm for 40 min; and (4) the MH2/MH2+ batches used for stability characterization were prepared with an initial temperature set point of 30 C, dispersion at 600–1000 rpm for 20 min, water addition over 45 min while increasing to 1500–2000 rpm, 60 min at the final speed, and sodium-benzoate solution added over 20 min after the main water addition. Because these datasets differ in processing, results are reported within each dataset and are not used to isolate a stirring-speed effect. The actual temperature, temperature rise, equipment model, rotor and vessel dimensions, and exact batch volume were not recorded, which limits exact reproduction.

Stability criteria: Visual stability was defined as the absence of a visible bulk phase boundary after the stated standing time. The actual ambient storage temperature was not recorded, and centrifugation is reported in rpm and time rather than relative centrifugal force because the rotor radius was unavailable.

Step 2: investigate the motion of the oil film on the steel plate using visualization techniques: Water and a transparent thickened resin solution with viscosity similar to that of fresh concrete slurry (flow value: 220~240 mm) were adopted to simulate concrete slurry separately. Invert the solution onto the formwork (stained oil film), observe the behaviour of the base oil on the formwork, and identify the base oil.

Step 3: After the 7 d visual check [22], stability was tested by high-speed centrifugation at 7000 rpm for 6 min; MH2+ was centrifuged for an additional 20 min. Visible phase boundaries were recorded. Optical microscopy was used as a qualitative morphological check.

Step 4: Demoulding performance was evaluated by a small-scale pull-off test and by photographs of the demoulded surfaces. The emulsion was diluted to 6.25 wt% non-volatile content, mixed with 1 wt% Dow CF-10 relative to the diluted sample, and brush-applied to a 100 mm × 100 mm 304 stainless-steel plate with nominal Ra = 1.6 micrometres. The applied wet mass was weighed, and the nominal oil-film thickness was calculated as delta_th = (m_wet × C_non-volatile)/(rho_dry × A), where m_wet is the applied wet mass, C_non-volatile is the non-volatile mass fraction of the diluted sample, rho_dry is the dry-phase density estimated from the component densities, and A is the nominal plate area. A 500 g portion of P.I 42.5 cement paste (water-to-cement mass ratio 0.29; GK-3000 admixture adjusted to a flow of 200–220 mm according to GB/T 8077-2012 [23]) was placed in a 300 mL plastic cup and inverted onto the horizontal coated plate. The assembly was cured at 20 +/− 2 C and 50–60% relative humidity for 18 h. The maximum separation force was recorded at a loading rate of 10 mm/min after a 1 N preload applied at 10 mm/min. Two records were available per formulation in the submitted table. They are reported individually with descriptive ranges, without standard deviation or significance testing. Photographs were taken under laboratory lighting and used qualitatively to identify visible streaks, pores and large-scale differences.

## 4. Results and Discussion

### 4.1. Emulsification of Soybean Oil

#### 4.1.1. Determination of the HLB Value Required for Emulsifying Soybean Oil

The chemical composition of soybean oil primarily consists of triglycerides, vitamins, and minerals, with triglycerides accounting for 90~95% of the total volume of soybean oil. Fatty acids are key constituents of triglycerides. Each triglyceride molecule is an ester compound formed by one glycerol unit and three fatty acid chains. Among the fatty acids in soybean oil, linoleic acid accounts for approximately 52~65%; oleic acid accounts for approximately 25~36%; palmitic acid accounts for approximately 6~8%; stearic acid accounts for approximately 2~5%; and linolenic acid accounts for approximately 2~3%. In addition, phospholipids account for approximately 1~3% of the total weight of soybean oil.

**Figure 1:**
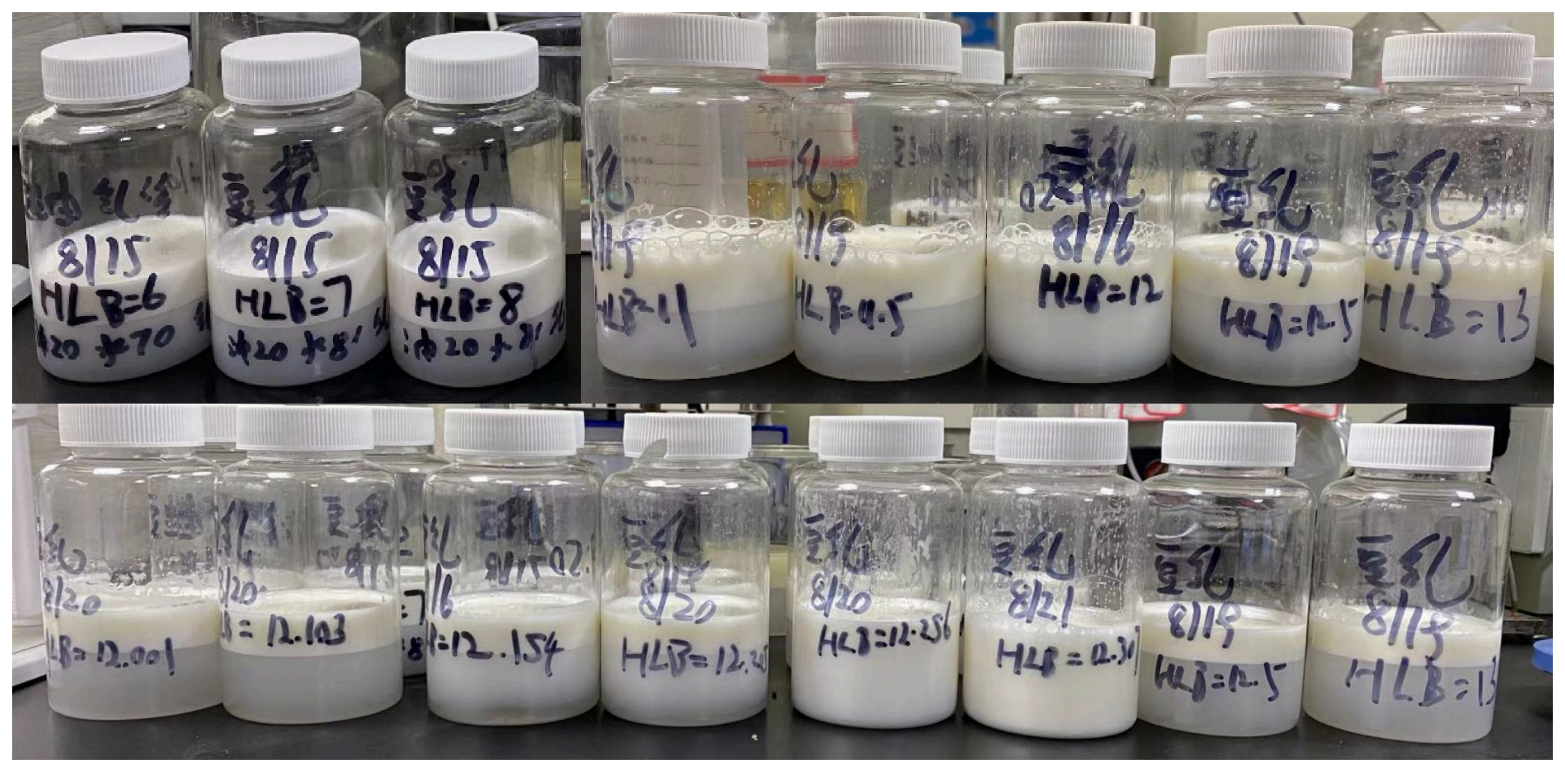
Phase separation in a soybean-oil emulsion (Span 80-OP10 emulsification system).

In the initial HLB screen, 20 g soybean oil, 5 g emulsifier and 80 g water kept the non-volatile fraction constant across conditions (this 20:5:80 preliminary ratio was not the final high-solids formulation). HLB was used as the emulsifier-selection criterion. Because literature values for the required HLB of soybean oil vary, Span-80 (HLB = 4.3) and OP-10 (HLB = 13.3–14.5) were blended to obtain different calculated HLB values (since OP-10’s HLB value is a range, 14.5 was used for calculations in this experiment). Emulsification used a high-speed homogenizer at 4000 rpm and an agent-in-oil route. Water was added dropwise over 20 min, followed by 20 min of emulsification and dispersion. The emulsion was then left at ambient laboratory temperature to assess separation at different HLB values. The formulations and calculated HLB values are summarized in Table 2, and the phase-separation results are shown in Figure 1.

Experiments showed that composite-emulsifier HLB values of 6, 7 and 8 produced visible separation within 30 min, whereas the nominal HLB 12 formulation showed only slight separation after 1 h. Screening between HLB 11 and 13 indicated a practical optimum in the 12.0–12.5 interval, and the fine screen showed only trace or small separation within 1 h across the calculated interval 12.15–12.31 under the assumption HLB(OP-10) = 14.5, with no visible bulk phase boundary. Because OP-10 itself is reported over 13.3–14.5, this number is not treated as a precise soybean-oil constant. The supported statement is a practical OP-10/Span-80 window of approximately 12.2–12.3 at the stated assumption, or approximately 11.3–12.3 when the lower and upper assumed OP-10 values are included.

HLB sensitivity: Because OP-10 does not have a single fixed HLB value, the value 12.26 is only a calculated coordinate under the explicit assumption HLB(OP-10) = 14.5. At the same 78:22 OP-10/Span-80 ratio, the calculated HLB values are 11.32, 11.79 and 12.26 for assumed OP-10 values of 13.3, 13.9 and 14.5, respectively (Table 3). The defensible description is therefore an emulsifier-system-specific range of approximately 11.3–12.3, with 12.2–12.3 giving the best visual result in the OP-10/Span-80 trials. This is not a thermodynamic HLB constant for soybean oil. For formulation work, however, the 12.2–12.3 interval provides a practical starting point for the OP-10/Span-80 system and reduces the number of emulsifier combinations that must be tested.

Table 2 reports mass fractions of the emulsifier blend. Its nominal HLB labels were calculated from the rounded blend fractions using HLB(OP-10) = 14.5 and HLB(Span-80) = 4.3. The fine-screen entries use the calculated value rather than the nominal label. This distinction is now stated to avoid treating 12.26 as an independently measured material property.

**Figure 2:**
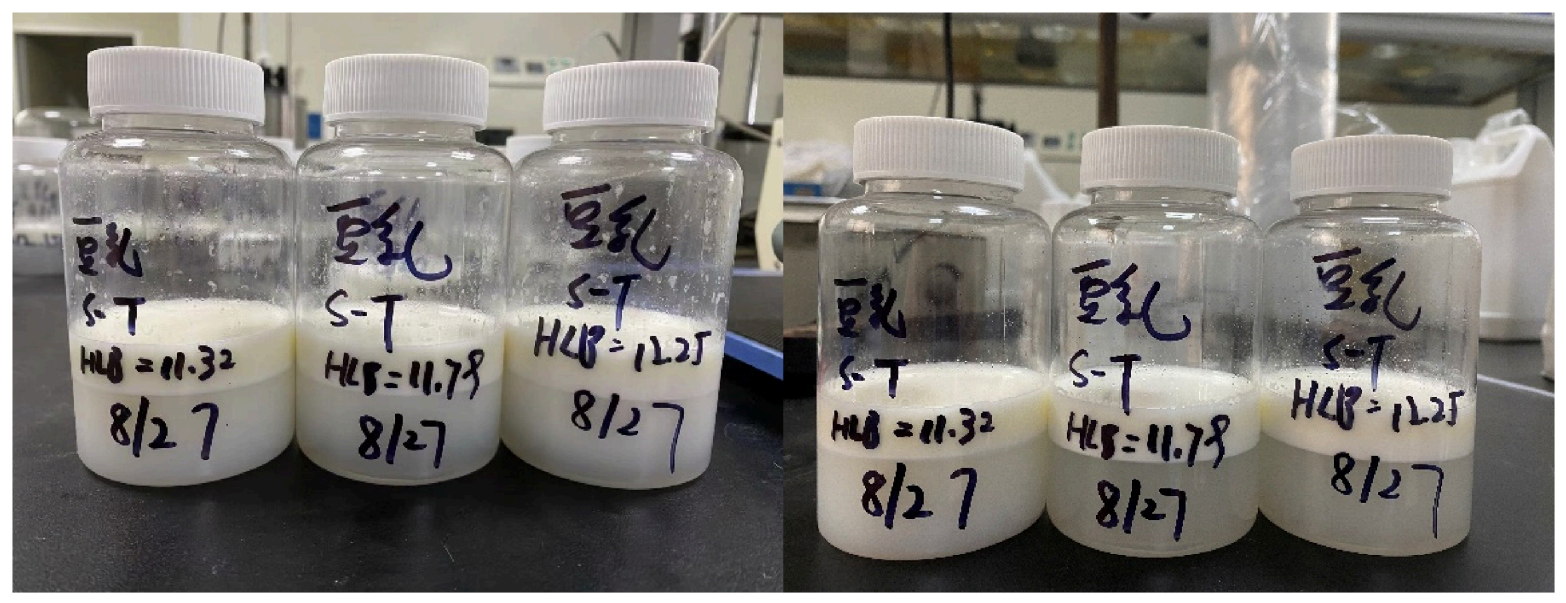
Phase separation in a soybean-oil emulsion (Tween 80–Span 80 emulsification system).

As the HLB of OP 10 covers a value range, the HLB results derived above lack high precision and cannot provide reliable references for switching to other emulsifiers. Therefore, Tween-80 with a fixed HLB value (HLB = 15) was purchased to replace OP-10 and be used in combination with Span 80 for emulsification. Based on the previous blending ratio of 78%:22% between OP-10 and Span 80 at an HLB value of 12.26, new HLB values were calculated (Table 3). Emulsification was then performed using the Tween-80/Span-80 emulsification system based on these new HLB values, and the resulting phase separation was observed (as shown in Figure 2).

As shown in Figure 2, obvious phase separation occurred on the day after emulsification for all samples, and their stability was inferior to that of the emulsion prepared with the optimal HLB value in the OP-10-Span 80 system. The emulsion with an HLB of 11.32 separated into two phases, while the emulsions with HLB values of 11.79 and 12.26 clearly exhibited three phases, with the bottom layer showing high transparency. After standing for one month, the emulsion with an HLB of 11.32 remained in the same state as the previous day, whereas the transparency of the bottom layer in the other two samples had increased noticeably. Two conclusions can be drawn from the above experiment:

1. Under the tested conditions, the OP-10/Span-80 system gave the best visual result near a calculated HLB of 12.2–12.3 when HLB(OP-10) = 14.5 was assumed. The Tween-80/Span-80 system was tested at the matching calculated HLB coordinates of 11.32, 11.79 and 12.26 (Table 3). These are emulsifier-system-specific screening coordinates, not universal HLB constants for soybean oil.

2. The Tween-80/Span-80 system was visually less stable than the OP-10/Span-80 system at the matched calculated HLB coordinates [24]. The result indicates that stability depends on the emulsifier system as well as on the HLB coordinate. A benzene-ring, interfacial-packing or film-elasticity explanation is plausible, but none of these features was measured directly. The statement therefore remains a hypothesis rather than a demonstrated cause.

#### 4.1.2. Emulsification Equipment and Process Trials

**Figure 3:**
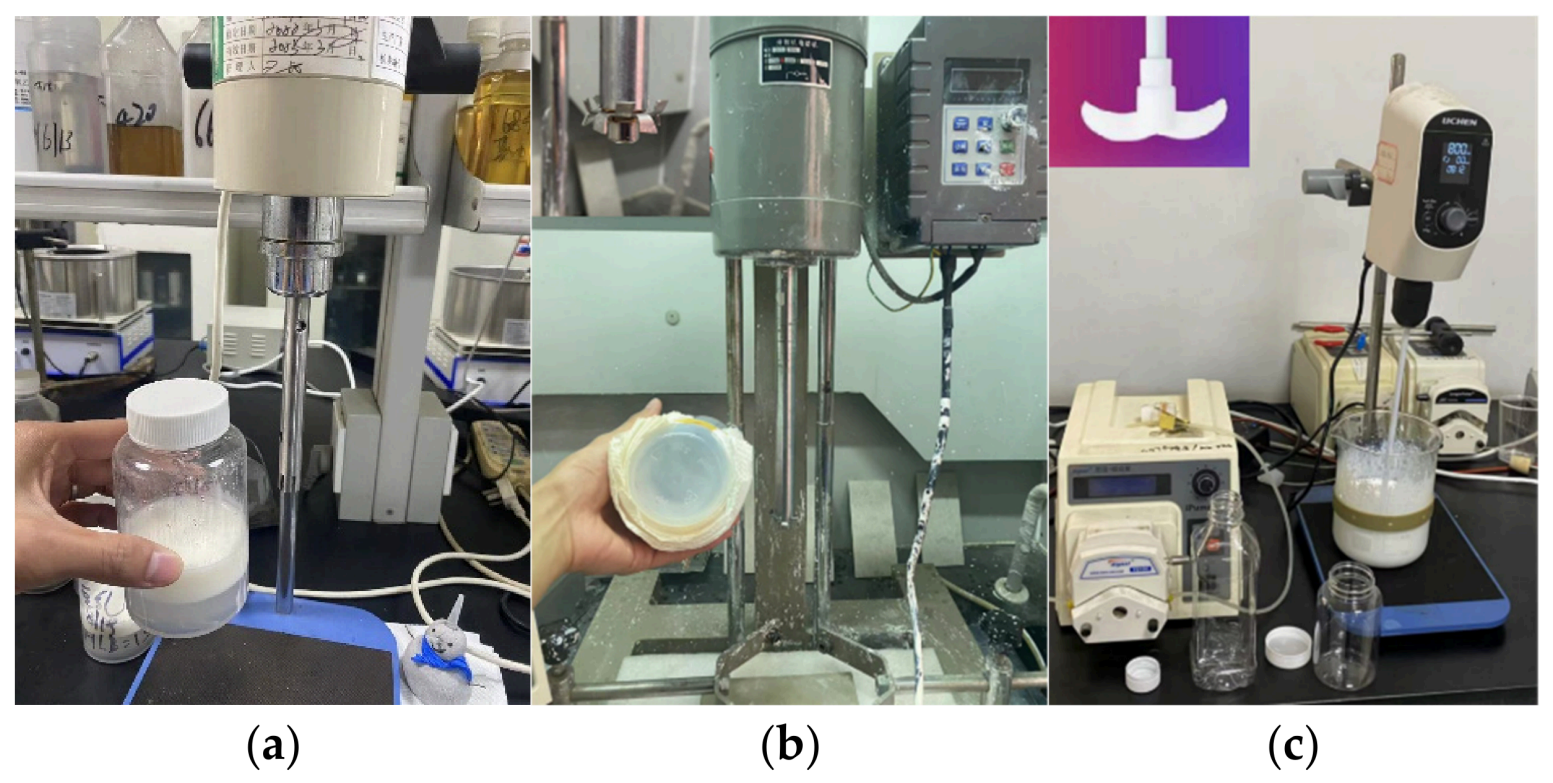
Different emulsification and mixing processes and equipment: (**a**) High-speed homogenizer; (**b**) High-speed disperser; (**c**) Electric mixer.

All preliminary HLB experiments were conducted using a high-speed homogenizer (Figure 3a). The main component of the homogenizer is the vessel, which contains a rotor designed to continuously circulate and break up the mixture. During operation, the rotor generates high-speed vortices and propels the liquid into the suspension. The cylinder also has an inlet and an outlet. The inlet is used to feed the mixture into the cylinder, and the outlet is used to discharge the processed mixture. Nevertheless, as illustrated in the figure, the working chamber of the homogenizer has a far smaller diameter than the reaction vessel. Hence, high emulsification efficiency cannot be guaranteed even at 4000 rpm. Therefore, further experiments were conducted using a high-speed disperser and an electric stirrer (Figure 3b,c). The emulsification mechanism of high-speed dispersers and electric stirrers primarily relies on the interaction between the high-speed rotating rotor and stator. Through the high tangential velocity and high-frequency mechanical effects generated, these devices exert a combination of intense shearing, centrifugal compression, inter-liquid friction, impact tearing, and turbulence on the material, thereby mixing immiscible liquids into an emulsion.

**Figure 4:**
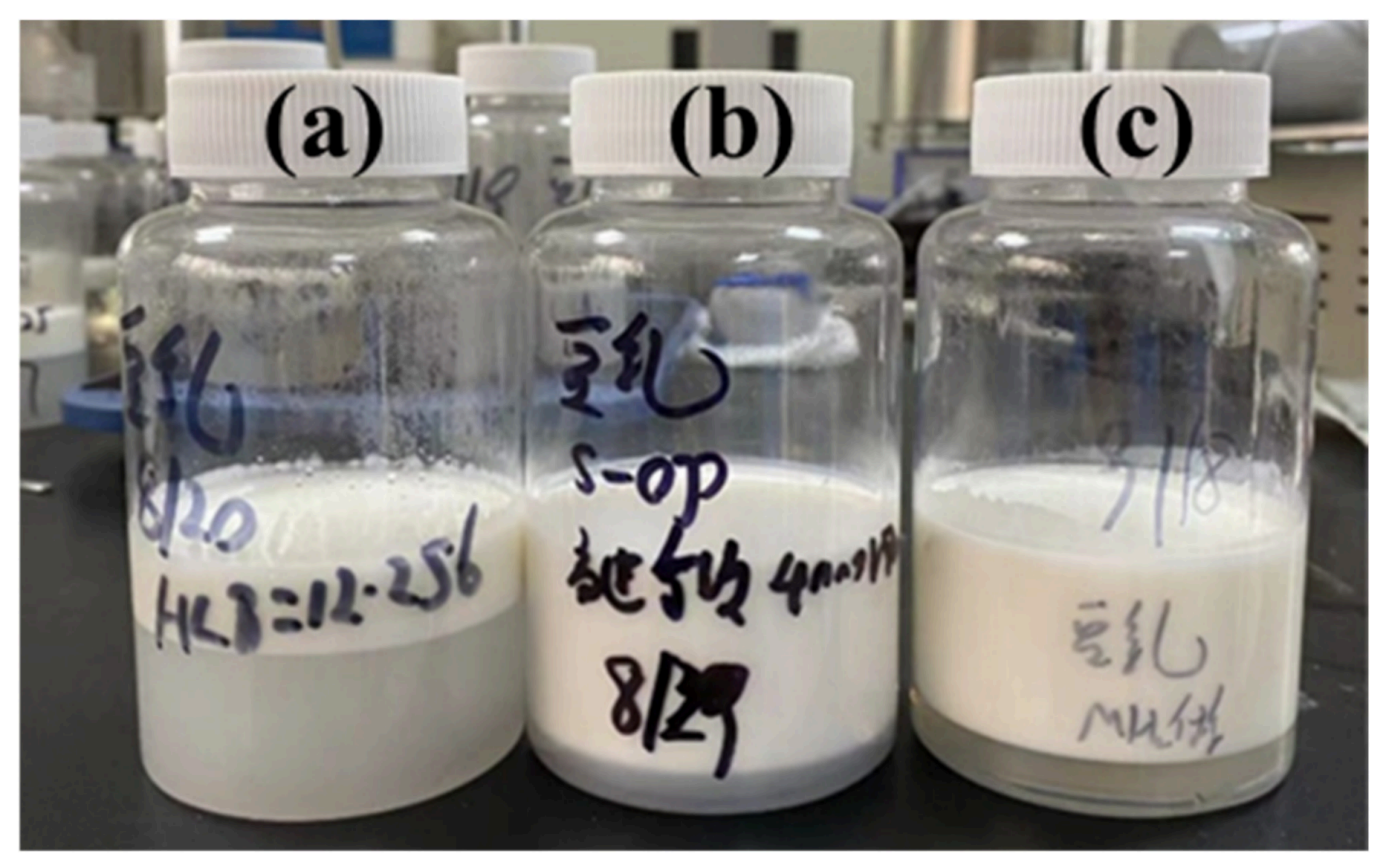
Soybean-oil emulsions prepared using different emulsification equipment: (**a**) High-speed homogenizer; (**b**) High-speed disperser; (**c**) Electric stirrer.

The three samples shown in Figure 4 are soybean-oil emulsions prepared using a homogenizer, a disperser and a stirrer. The homogenizer and disperser samples contained 19% soybean oil, 4.8% blended emulsifier and 76% deionized water. After mixing, the sample was dispersed at 4000 rpm for 5 min. Water was added dropwise over 20 min and dispersion continued for another 20 min. After nearly one month at ambient laboratory temperature, the disperser sample showed better visual resistance to separation than the homogenizer sample. A plausible explanation is that the disperser provides a wider shear region and more droplet breakage; because the equipment geometry and operating conditions also differed, the difference is not attributed to rotor type alone.

The high-oil-phase soybean-oil emulsion used in Figure 4c contained 48.36 wt% soybean oil, 2.14 wt% OP-10, 2.14 wt% Span-80, 2.96 wt% oleic acid, 1.00 wt% sodium benzoate and 43.40 wt% deionized water (100.00 wt% in total; 41.11 wt% water was added directly and 2.29 wt% was used to dissolve sodium benzoate). The oil/emulsifier/oleic-acid mixture was dispersed at 600 rpm for 10 min. Main water was added dropwise and the sodium benzoate solution was added thereafter over a combined addition period of 45 min, followed by dispersion at 800 rpm for 40 min. The previously reported 48.0/4.3/2.9/1.0/44.5 wt% list summed to 100.7 wt% and has been corrected.

This comparison changes mixing speed together with oil fraction, emulsifier loading, oleic acid and sodium benzoate. It therefore establishes that the complete formulation could be processed at 600–800 rpm; because composition and processing time also changed, it is not used to isolate a speed effect. A factorial experiment at fixed composition and fixed processing time would be required to test the effect of stirring speed.

Possible explanations for the association between sodium benzoate and emulsion stability include changes in ionic strength, benzoate interaction at the interface and altered flocculation [25,26]. The present data are consistent with these possibilities but do not distinguish their relative contributions. Controlled pH and ionic-strength experiments, together with interfacial adsorption and viscoelasticity measurements, would be required to resolve the mechanism. From a practical standpoint, the complete high-non-volatile formulation can be prepared in a low-speed laboratory mixer, which supports laboratory-scale reproduction even though the effect of stirring speed alone was not isolated.

### 4.2. Qualitative Retention of Base Oils on Formwork Materials

**Figure 5:**
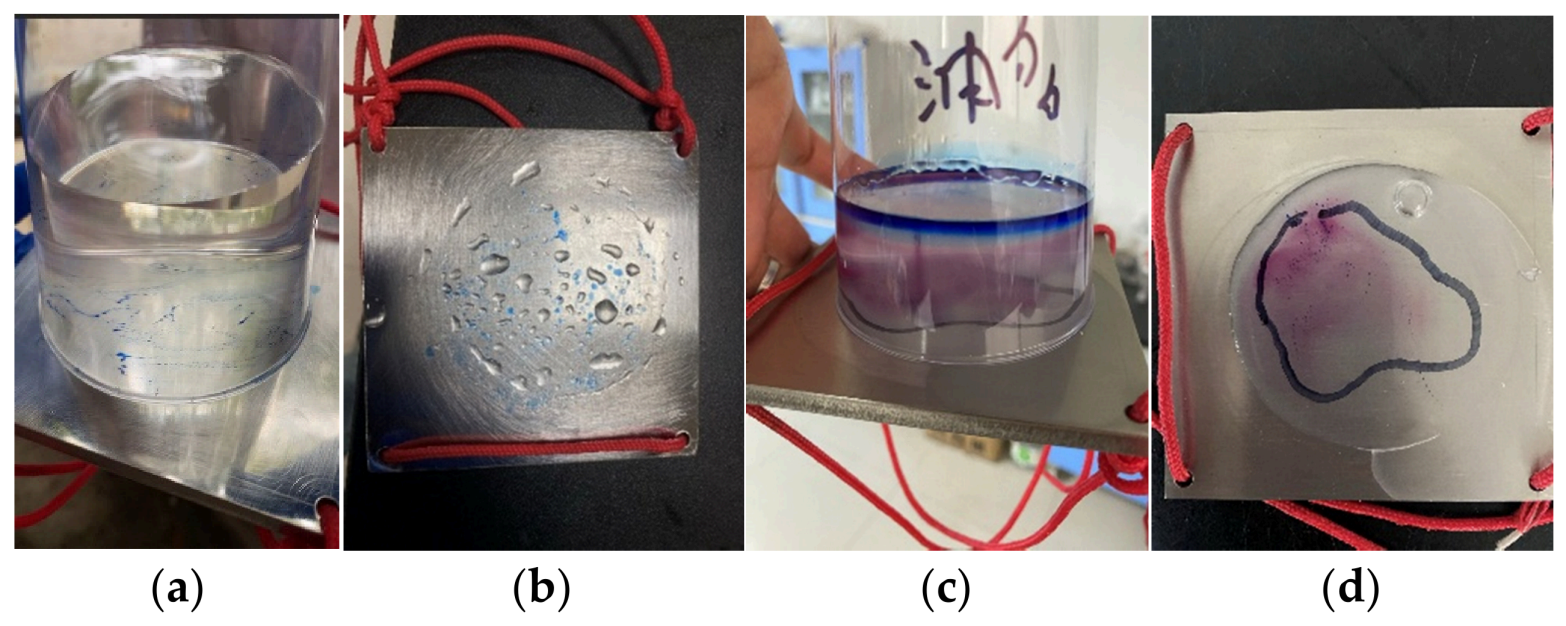
State transition of oil No. 46 in B3 (a high-viscosity cement pore fluid): (**a**) Visualization setup showing the interaction between a low dose of No. 46 low-viscosity mineral oil and B3; (**b**) Distribution of the oil film on a stainless-steel plate after 24 h of contact between a low dose of No. 46 low-viscosity mineral oil and B3; (**c**) Visualization setup showing the interaction between a high dose of No. 46 low-viscosity mineral oil and B3; (**d**) Distribution of the oil film on a stainless-steel plate after 24 h of contact between a high concentration of No. 46 low-viscosity mineral oil and B3.

Preliminary tests on No.46 low-viscosity mineral oil (40 °C kinematic viscosity: 44.10 mm^2^/s) revealed that at low oil dosage and under the effect of hydrophilic media, the oil shrank into unevenly distributed droplets. Furthermore, there were very few such droplets (resulting in a small effective oil film area), which prevented it from providing effective isolation—a factor that also contributed to mould sticking. When a larger amount of oil was present, some of the oil rose slowly as it was impacted by the hydrophilic medium. Although the ascent was slow due to viscous resistance, observation after 24 h revealed that a small amount of oil remained within the initially marked area (Figure 5).

**Figure 6:**
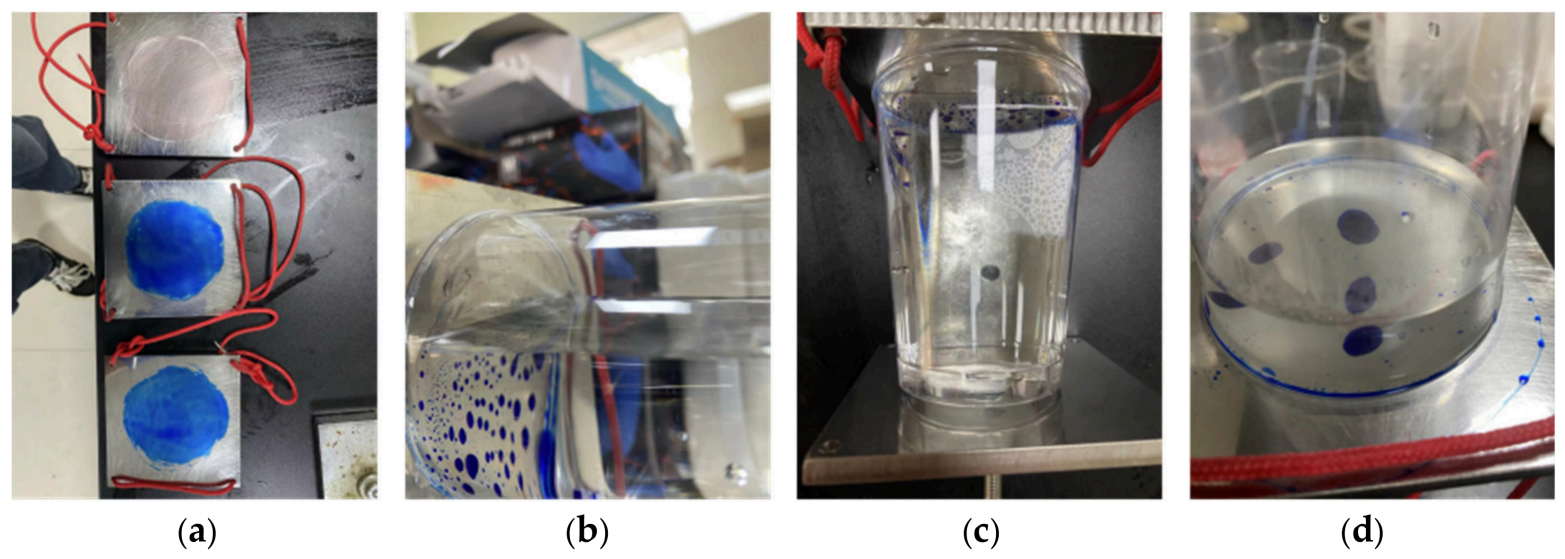
Affinity test of naphthenic oil and stainless-steel moulds: (**a**) Distribution of naphthenic oil on a stainless-steel plate; (**b**,**c**) Distribution of the oil film on a stainless-steel plate after 24 h of contact between the naphthenic oil and B3; (**d**) Distribution of the oil film on a stainless-steel plate after 48 h of contact between the naphthenic oil and B3.

The observed phenomenon with oil No. 46 may be caused by its low viscosity or low affinity with the formwork. Since concrete demoulding requires substantial amounts of oil-phase components to remain at the formwork interface to provide effective separation [27], we repeated the above test using high-viscosity 4010 naphthenic oil (viscosity at 40 °C: 157.2 mm^2^/s) and added formwork placed vertically on the side to simulate the side panels at a construction site. The results are shown in Figure 6. After the naphthenic oil was applied to the side formwork, upon contact with a hydrophilic medium, the oil droplets contracted and coalesced, but most remained adhered to the formwork surface, with only a small number floating upward. Meanwhile, the oil droplets on the base plate also merely contracted and did not float upward in large numbers as was the case with the No. 46 oil. This indicates that naphthenic oil was retained more strongly by stainless steel than by polystyrene in this simplified test.

**Figure 7:**
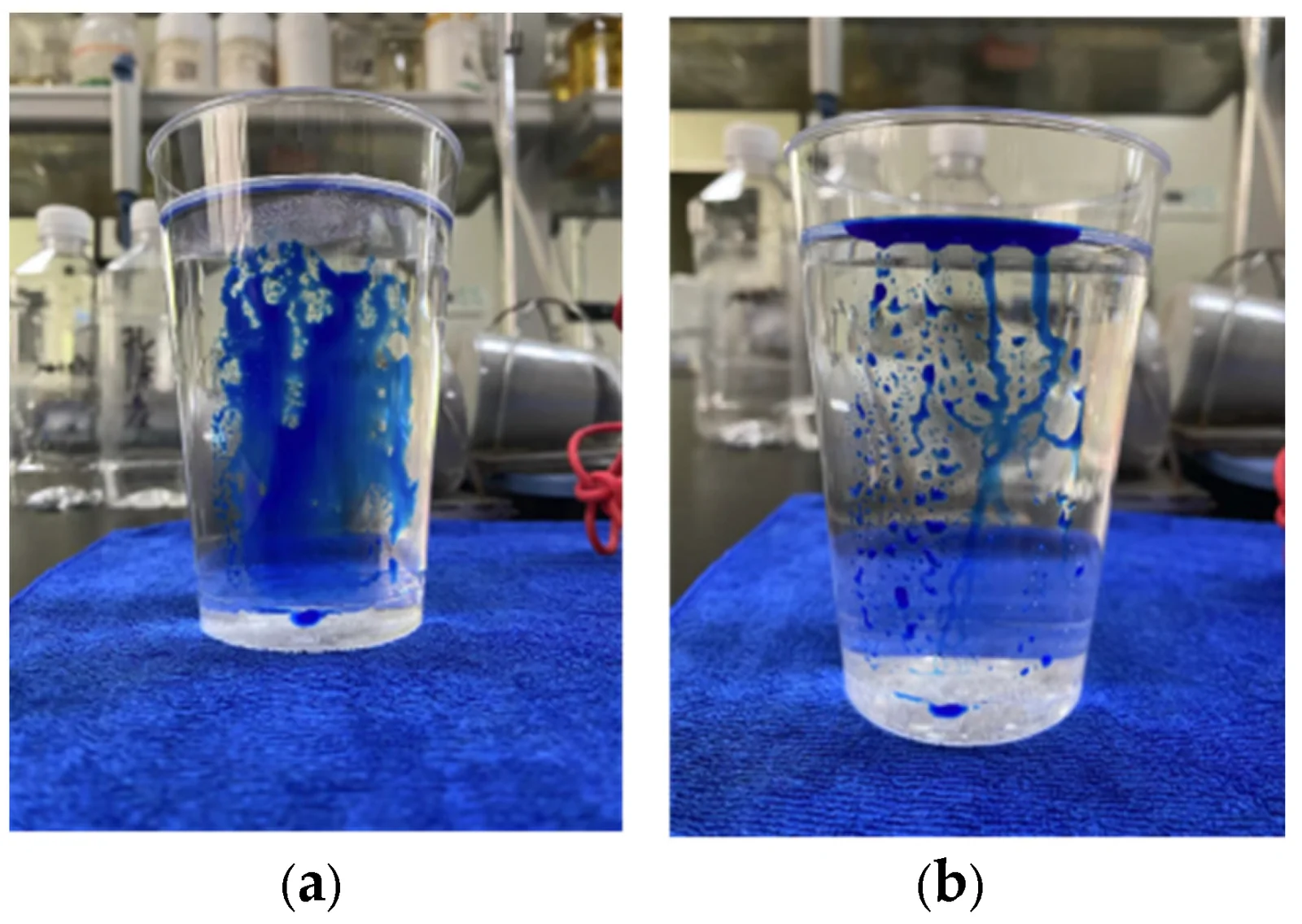
Affinity test of naphthenic oil and polystyrene templates: (**a**) 0 min of contact; (**b**) 60 min of contact.

Nevertheless, when a polystyrene cup was used as the mould substrate, most naphthenic oil floated upward within 1 h. In this test, retention on stainless steel was stronger than on polystyrene. The comparison is therefore limited to the tested surfaces and does not establish a general surface-energy rule. These observations are shown in Figure 7.

The affinity test is a simplified static retention observation. Water and the thickened resin solution approximate a hydrophilic medium, they do not reproduce fresh-cement chemistry, hydration, pressure, flow or vibration, so the result is limited to qualitative retention on the tested surfaces. The comparison between stainless steel and polystyrene also involves differences in surface energy, roughness and surface chemistry. The test is not a rain-washout measurement, and field performance under rain would require separate validation. For steel formwork, the result nevertheless supports selecting the higher-viscosity naphthenic oil over the low-viscosity mineral oil, because it remains more strongly retained under the simplified hydrophilic exposure.

### 4.3. Association of Sodium Benzoate Addition with MH2 Emulsion Stability

**Figure 8:**
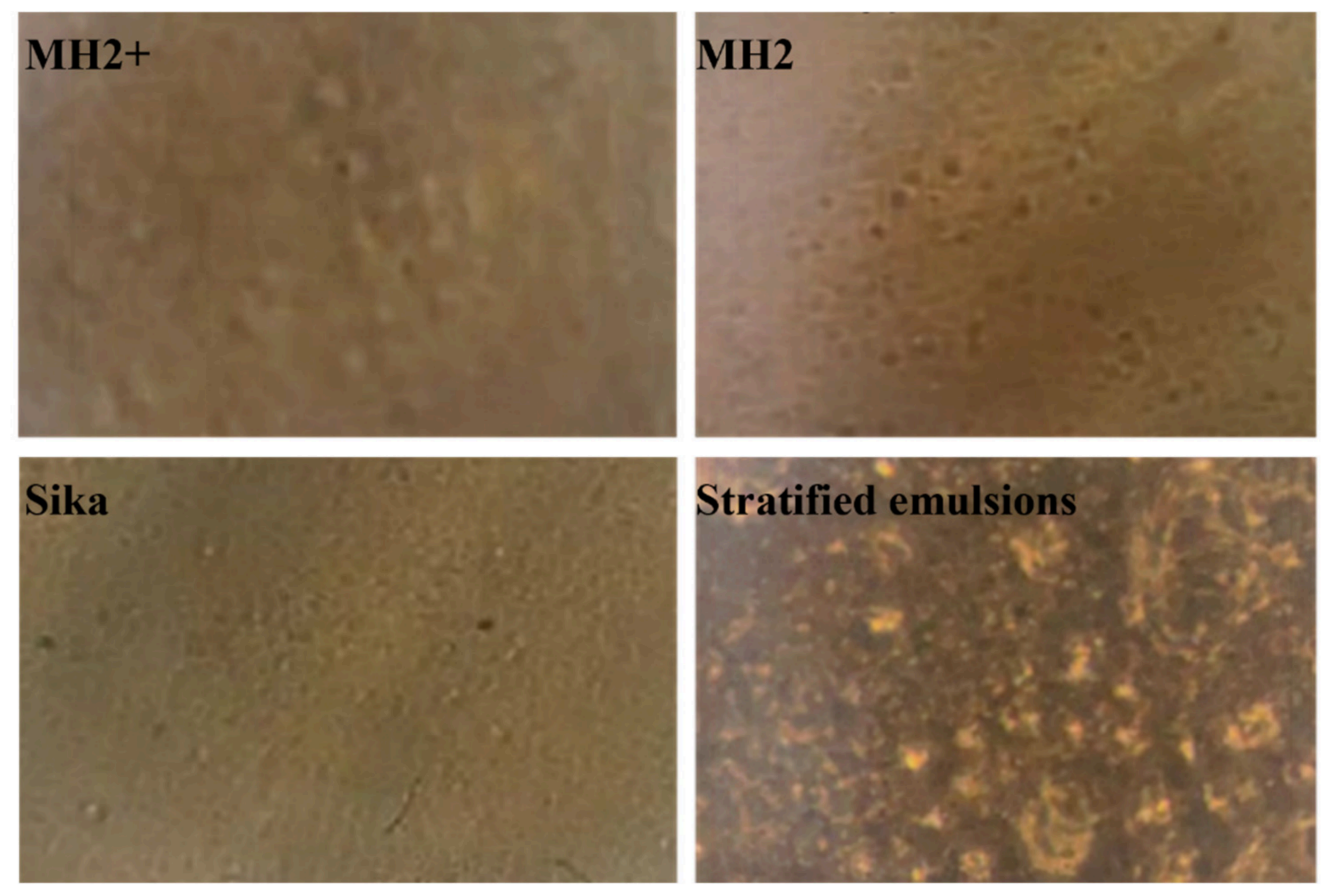
Qualitative optical-microscopy comparison of a commercial Sika product and the MH2 series (60 × 16 magnification).

Figure 8 compares the visual microscopic morphology of a commercial Sika product and the MH2 series only as a qualitative benchmark.

MH2 formed a fine bluish emulsion during preparation but still separated during storage. Optical microscopy alone cannot establish nanoscale dispersion or a size distribution, so the stability assessment is based on the observed phase separation and the specified centrifugation test. Previous drying studies show that substrate wettability affects the continuity of oil-in-water films [10], which provides a plausible context for the visual differences observed here.

**Figure 9:**
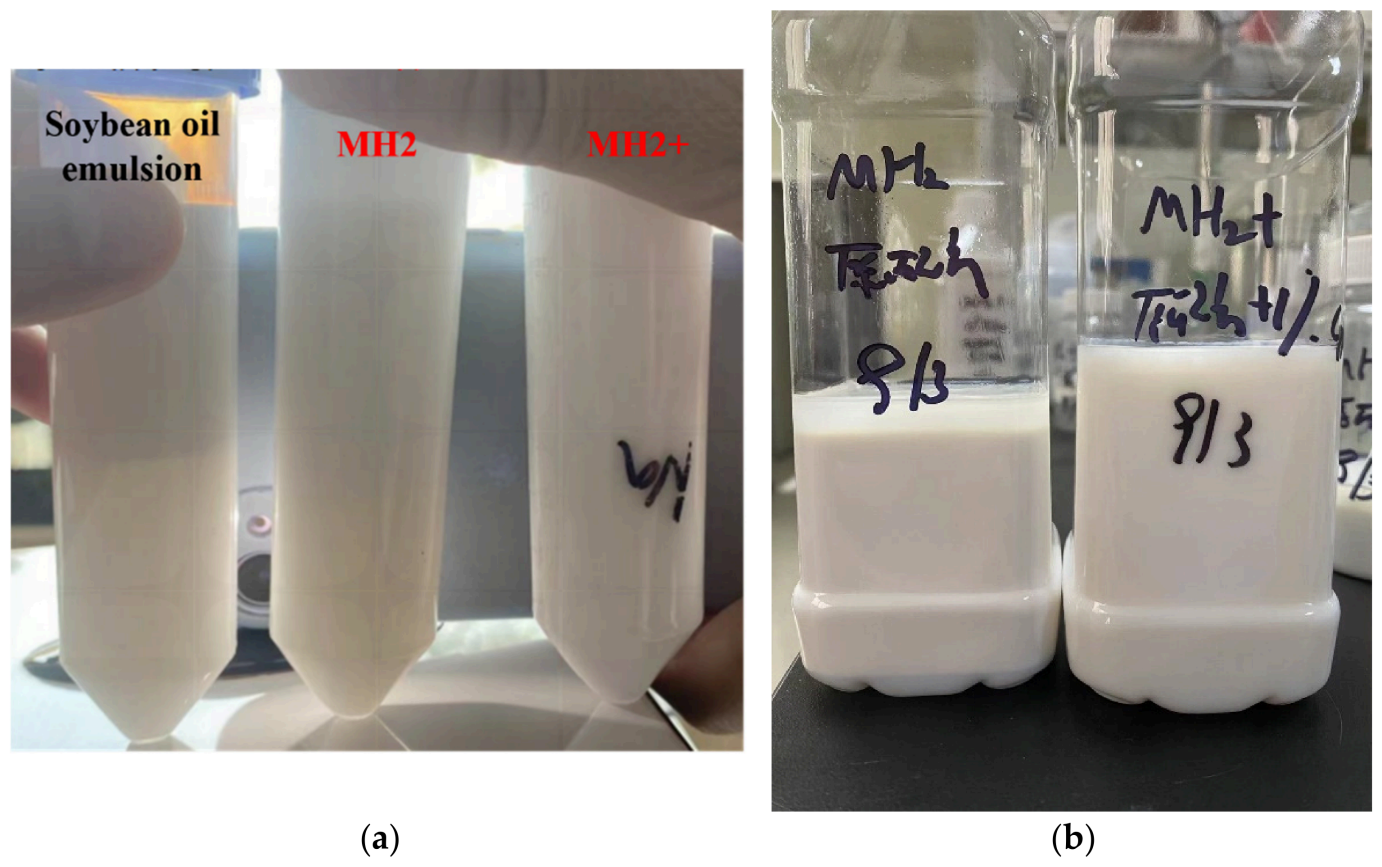
Emulsion separation after centrifugation (7000 rpm, 6 min) and after standing at room temperature: (**a**) After centrifugation; (**b**) After standing for 20 days.

As shown in Figure 9, after high-speed centrifugation, the MH2+ emulsion remained relatively stable without visible phase separation. While MH2 showed no sharp phase boundary comparable to the soybean-oil emulsion, its bottom layer possessed markedly higher light transmittance than the top layer. When the MH2+ emulsion was subjected to a further 20 min high-speed centrifugation, it still did not separate into phases. After being stored undisturbed at room temperature for 20 days, MH2 exhibited distinct three-phase separation, while a thin oil layer was also observed on the surface of MH2+. However, this oil layer re-emulsified upon the addition of water, suggesting that the interfacial film was not sufficiently tight or stable, and that some oil droplets had agglomerated and floated to the surface.

The quiescent-storage endpoint in the present submission is 20 d, not a shelf-life test. MH2 separated into three visible phases, whereas MH2+ formed a thin surface oil layer that re-emulsified when water was added. Stability in this paper therefore means the absence of a visible bulk phase boundary under the specified test and observation interval. Long-term stability is not claimed.

**Figure 10:**
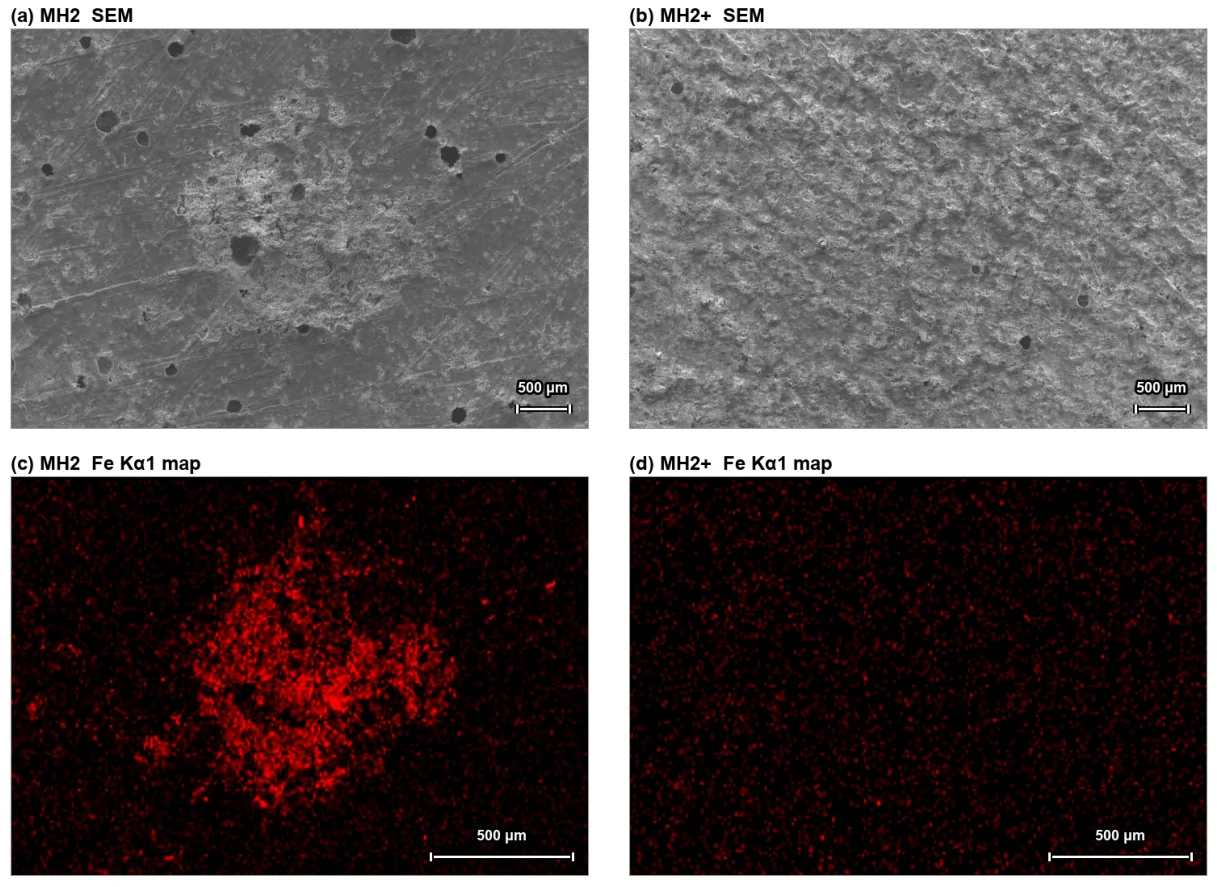
Representative SEM images and Fe elemental maps of the corrosion-related surfaces after demoulding: (**a**) MH2, SEM; (**b**) MH2+, SEM; (**c**) MH2, Fe map; (**d**) MH2+, Fe map. The SEM and EDX panels illustrate related MH2/MH2+ surface observations but were not spatially registered, and their fields of view differ. The elemental maps are qualitative; colour intensity is not a quantitative concentration scale.

Qualitative rust-transfer observation: Separate reused 304 stainless-steel plates with similar visible corrosion histories were wire-brushed, coated with MH2 or MH2+, and contacted with the same P.I 42.5 cement paste under the same curing conditions. After demoulding, the MH2-formed surface showed numerous rust-coloured spots, whereas the MH2+-formed surface showed no visible rust spots. EDX mapping detected local Fe enrichment on the MH2 surface but no comparable feature on MH2+. The reused plates had Ra = 3.2 micrometres, and the elemental maps were qualitative. This observation is consistent with the known corrosion-inhibiting function of sodium benzoate [20] and supports a role of the sodium-benzoate-containing formulation in reducing visible rust transfer; a corrosion rate and the relative contributions of inhibition and film continuity would require dedicated electrochemical testing. Figure 10 presents representative SEM images and Fe elemental maps from related surface observations.

The 1.00 wt% sodium benzoate dosage was selected as the formulation of interest. A concentration optimum and the individual role of oleic acid remain open questions for formulation optimization. Overall, the centrifugation and 20 d storage results identify MH2+ as the more storage-stable candidate in this system, while the molecular role of sodium benzoate remains to be resolved.

### 4.4. Preliminary Pull-Off Tests of Soybean-Oil Emulsion, MH2+ and Their 1:3 Blend

MH2 uses the OP-10/Span-80 system, which was also used for the soybean-oil emulsion. The emulsifier ratio for the soybean-oil emulsion was adjusted to the soybean-oil HLB window identified above. After oleic acid and sodium benzoate were added, the emulsion still separated but appeared visually more stable than the earlier samples. Separating the contributions of the two additives would require same-composition controls. The soybean-oil emulsion, MH2+, and their 1:3 mass blend were then compared with Jinghuada EP6 in preliminary pull-off tests and surface photographs.

Pull-off data treatment: The pull-off protocol is given in Step 4. The exact inner diameter of the 300 mL cup was not retained in the original record and is therefore not reconstructed. The contact area is defined by the full cup opening. The application method and target coating amount determine the deposited film thickness [28], and the pull-off force is used only as an engineering proxy for the formwork/fresh-concrete tribological interaction [14].

The reported oil-film thickness values in Table 4 are nominal application coordinates calculated from the applied wet mass, non-volatile mass fraction, dry-phase density and nominal plate area. They are process-control values, not local thickness measurements. The release test is a small-scale laboratory proxy and was not validated against a recognized demoulding-force standard.

For the submitted Table 4, only two records per formulation were available. They are therefore reported as individual values and a descriptive range; standard deviations and inferential tests are not calculated from n = 2. The commercial products were selected as availability-based benchmarks: Sika Separol W-306 for a manufacturer-stabilized microscopic comparison and EP6 for the application-level pull-off comparison. They are not presented as a representative sample of the commercial market.

At closely matched nominal thicknesses of 1.57–1.59 micrometres, the 1:3 blend, MH2+ and the soybean-oil emulsion gave 7.92, 11.63 and 55.26 N, respectively. This narrow-range comparison supports a lower force for the blend at sufficient coating, but the blend increased to 73.68 N at 1.05 micrometres, indicating strong sensitivity to coating amount and possible film discontinuity. Because the two blend specimens had different thicknesses, a replicated thickness series would be needed to separate thickness, film uniformity and formulation effects. The other soybean-oil record was reported only as ‘200+’; it is a censored record above 200 N and is excluded from finite-value comparison. MH2+ gave 10.36 and 11.63 N at 1.48 and 1.59 micrometres, with a descriptive mean of 11.0 N and range of 10.36–11.63 N. EP6 gave 7.963 and 10.326 N at 1.50 and 1.70 micrometres, with a descriptive mean of 9.14 N and range of 7.963–10.326 N. These means and ranges are descriptive and are not used for inferential ranking.

Table 4 note: each row is one pull-off record; two records were available per formulation. ‘200+’ is a right-censored value above 200 N and is not a finite force. No standard deviation or significance test is reported.

**Figure 11:**
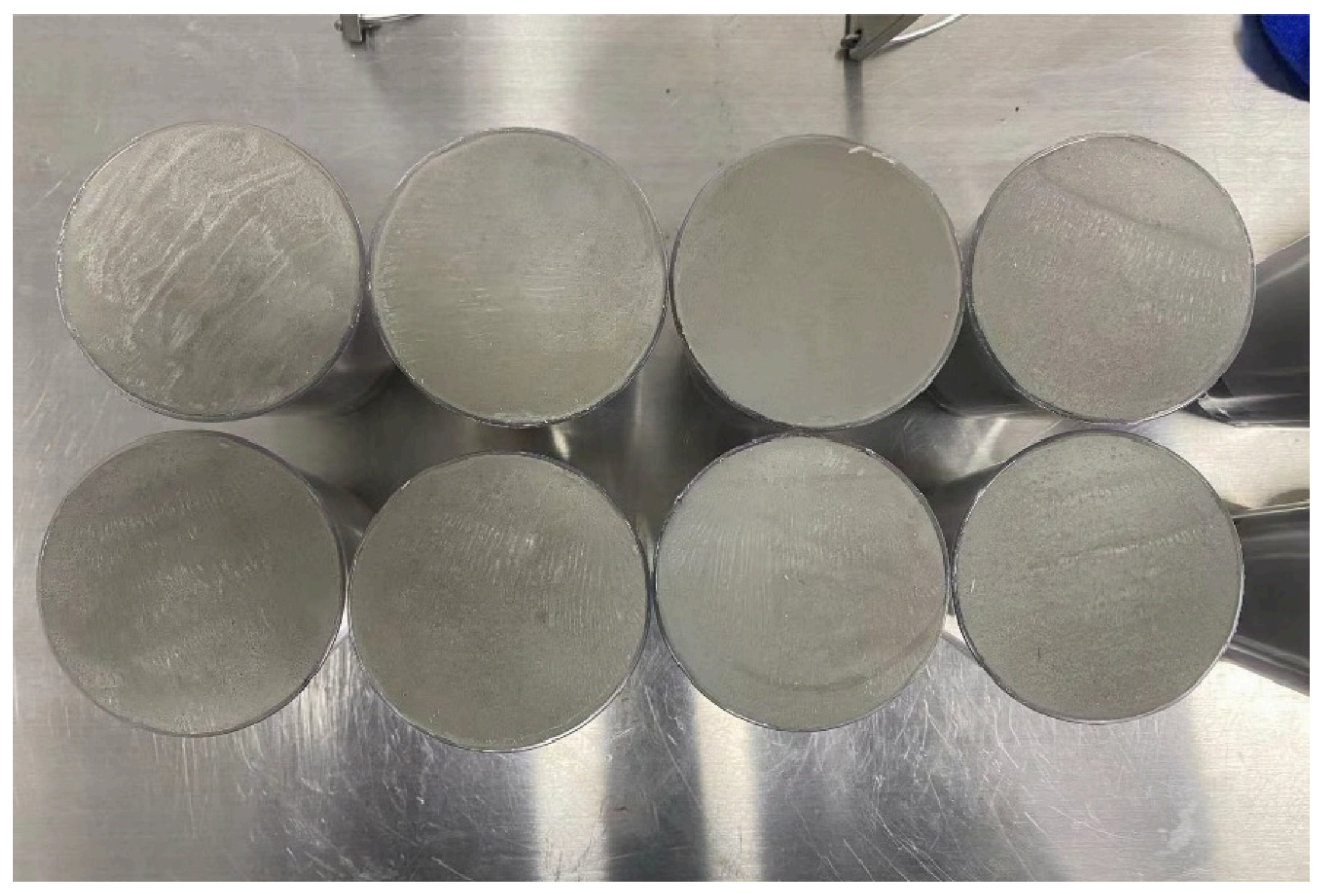
Demoulding appearance with different release agents (from left to right: soybean-oil emulsion; MH2+; a 1:3 mixture of soybean-oil emulsion and MH2+; EP6).

The tests revealed that the soybean-oil emulsion produced an uneven oil-phase distribution after film formation. In Figure 11, the thicker top-left oil layer showed white streaks. MH2+ gave 10.36–11.63 N near a nominal thickness of 1.5 micrometres, close to the EP6 observations of 7.963–10.326 N near 1.5–1.7 micrometres. According to N-B-19305 [29], the 1:3 blend produced a visibly more uniform, less streaked surface than the stand-alone soybean-oil emulsion, indicating an improvement in colour uniformity under the tested conditions. This result is consistent with the findings of Danuta et al. [30]. Its pull-off force nevertheless ranged from 7.92 to 73.68 N across the two available records; at a comparable thickness, the 7.92 N value was comparable to the 7.963 N recorded for EP6 at 1.50 micrometres. The image is recorded as a qualitative result. In this result, the combination of a pull-off force comparable to EP6 and a more uniform surface makes the 1:3 blend the most promising wax-free formulation tested at sufficient coating.

## 5. Conclusions

In this work, soybean-oil emulsification was studied and a wax-free water-based soybean-oil/naphthenic-oil release agent was prepared and evaluated. Using OP-10/Span-80 blends, the best visual stability was obtained in a calculated HLB window of 12.15–12.31 at an assumed OP-10 HLB of 14.5, which provides a practical formulation window of about 12.2–12.3 and reduces the number of emulsifier combinations to be screened. The complete high-non-volatile formulation could be prepared in a low-speed laboratory mixer by adding water to the oil phase; this is a practical laboratory preparation route, although the experiments did not isolate the effect of stirring speed. Adding 1.00 wt% sodium benzoate (MH2+) was associated with better visual and centrifugal storage stability than the sodium-benzoate-free emulsion and with the absence of visible rust transfer in the qualitative reused-plate test, while the molecular mechanism remains unresolved. Oleic acid was used as the emulsifying aid, although its individual contribution was not isolated. At nominal thicknesses of 1.57–1.59 micrometres, the 1:3 blend of soybean-oil emulsion and MH2+ gave a pull-off force of 7.92 N, comparable to EP6 (7.963 N at 1.50 micrometres) and lower than MH2+ (11.63 N) and the soybean-oil emulsion (55.26 N), and it produced a visually more uniform demoulded surface, making it the most promising wax-free formulation tested at sufficient coating. Its force increased to 73.68 N at 1.05 micrometres, so the coating amount must be controlled, and the two available records per formulation mean that the pull-off results are descriptive. The higher-viscosity 4010 naphthenic oil also showed stronger retention on stainless steel than the low-viscosity mineral oil and polystyrene in the simplified affinity test, supporting its selection for steel formwork. These results establish a practical formulation window, a feasible low-speed preparation route and a promising low-force blend, while rain washout, quantitative corrosion inhibition, concrete strength, coating adhesion and field durability require separate validation.

## Figures and Tables

**Table 1:** Experimental emulsifiers and their properties.

Emulsifier	Structural Formula	HLB	Molecular Weight
Oleic Acid (CH_3_(CH_2_)_7_CH=CH(CH2)_7_COOH)	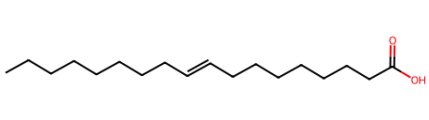	1	282.46
Span-80(C_24_H_44_O_6_)	Dehydrated sorbitol monooleate 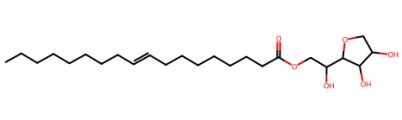	4.3	429
OP-10 (C_15_H_24_O·(C_2_H_4_O) _n_)	Nonylphenol polyoxyethylene ether-10, ethylene oxide condensate according to the number of EO is divided into OP-4, OP-6, OP-7, OP-8, OP-10, OP-12, OP-15, etc. 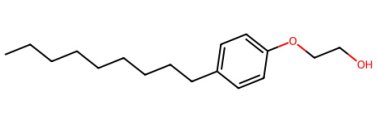	13.3~14.5	
Tween-80 (C_24_H_44_O_6_(C_2_H_4_O) _n_)	Polyoxyethylene dehydrated sorbitol monooleate 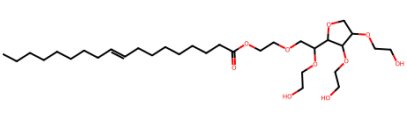	15	
CO-40	Polyoxyethylene hydrogenated castor oil, hydrogenated castor oil and ethylene oxide condensate, according to the number of EO is divided into 40,60,80, etc.	13~14	
EL-40	Castor oil polyoxyethylene ether is divided into EL-10, EL-20, (H) EL-40, EL-60, EL-80 and so on according to the number of EO. The structural formula of castor oil polyoxyethylene ether can be expressed as follows: R-CO-O-(CH2CH2O) n-H Among them, R represents the fatty alcohol part of castor oil, and n represents the degree of polymerization of polyoxyethylene ether.	13~14	
Tripolyglycerol monostearates (C_27_H_54_O_8_)	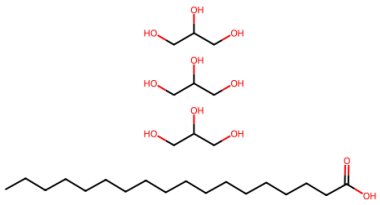	7	506.72
Tripolyglycerol monooleate (C_27_H_52_O_8_)		7~8	504.7
Decapolyglycerol monooleate (C_36_H_70_O_14_)		13.5~14	726.9
Soybean lecithin (60%) (C_12_H_24_NO_7_P)	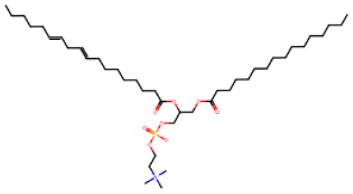	14	759.1
Sodium dodecyl sulphate (C_12_H_25_SO_4_Na)	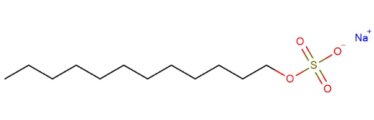	40	288.379

**Table 2:** Soybean-oil emulsifier formulations and calculated HLB values for the OP-10/Span-80 system.

Calculated HLB (OP-10 = 14.5)	OP-10 Mass Fraction in Emulsifier	Span-80 Mass Fraction in Emulsifier	Visual Observation
6.03 (nominal 6)	17%	83%	Noticeable separation within 30 min
7.05 (nominal 7)	27%	73%	Noticeable separation within 30 min
8.07 (nominal 8)	37%	63%	Noticeable separation within 30 min
11.13 (nominal 11)	67%	33%	Noticeable separation within 1 h
12.15 (nominal 12)	77%	23%	Trace-level separation within 1 h
13.17 (nominal 13)	87%	13%	Noticeable separation within 1 h
11.50 (fine)	71%	29%	Slight separation within 1 h
11.90 (fine)	74.5%	25.5%	Slight separation within 1 h
12.00 (fine)	75.5%	24.5%	Slight separation within 1 h
12.10 (fine)	76.5%	23.5%	Slight separation within 1 h
12.15 (repeat)	77%	23%	Trace-level separation within 1 h
12.21 (fine)	77.5%	22.5%	Trace-level separation within 1 h
12.26 (fine)	78%	22%	A small amount of clear liquid has settled at the bottom
12.31 (fine)	78.5%	21.5%	Slight separation within 1 h
12.56 (fine)	81%	19%	Slight separation within 1 h

**Table 3:** Formulation of soybean-oil emulsifiers (Tween-80/Span-80 emulsion system).

Assumed HLB Value for OP-10	HLB	Tween-80 Content in Emulsifiers	Span-80 Content in Emulsifiers
13.3	11.32	65.6%	34.4%
13.9	11.79	70%	30%
14.5	12.26	74.3%	25.7%

**Table 4:** Pull-off forces for different release agents.

Pull-Off Test	Category	Nominal Oil-Film Thickness/Micrometres	Maximum Pull-Off Force/N
Template roughness, Ra = 1.6 μm Non-volatile content 6.25%	Soybean-Oil Emulsion	1.68	200+
		1.58	55.263
	MH2+	1.59	11.634
		1.48	10.36
	Soybean-Oil Emulsion/MH2+ = 1:3	1.05	73.68
		1.57	7.92
	EP6	1.50	7.963
		1.70	10.326

## Data Availability

The processed data underlying Table 4 and the reported stability indicators are included in this article. Raw pull-off records and nominal film-thickness calculations are available from the corresponding author on reasonable request because the original laboratory records are not in a reusable electronic format.
